# Identification of the NADPH Oxidase (Nox) Subtype and the Source of Superoxide Production in the Micturition Centre

**DOI:** 10.3390/biology11020183

**Published:** 2022-01-24

**Authors:** Qin Wu, Ayse Gurpinar, Maxwell Roberts, Patrizia Camelliti, Michael R. Ruggieri, Changhao Wu

**Affiliations:** 1 School of Medicine, Jiangsu Vocational College of Medicine, Yancheng 224005, China; hhwuq@163.com; 2 School of Biosciences and Medicine, University of Surrey, Guildford GU2 7XH, UK; a.gurpinar@surrey.ac.uk (A.G.); m.w.roberts@surrey.ac.uk (M.R.); p.camelliti@surrey.ac.uk (P.C.); 3 Department of Anatomy & Cell Biology, Temple University, Philadelphia, PA 19122, USA; michael.ruggieri@temple.edu

**Keywords:** NADPH oxidase (Nox), periaqueductal gray (PAG), pontine micturition centre (PMC), reactive oxygen species (ROS), bladder, aging

## Abstract

**Simple Summary:**

Reactive oxygen species (ROS) are chemically active oxygen-containing molecules and overproduction of ROS can cause oxidative damage to cells and tissues in the body. Oxidative damage to brain cells can not only cause lesions to the brain but also lead to disorders in peripheral organs under the control of the corresponding brain centres, such as the urinary bladder. A unique class of enzymes that produce ROS are the special oxidising enzymes called “Nox” enzymes. These are the body’s only enzymes that can be selectively controlled without affecting normal cell activity. Therefore, Nox enzymes are considered to be a drug target. Whether Nox exists in the brain centres that control urination has not been examined. We investigated whether the type 2 Nox enzyme-Nox 2 exists in the brain urination control centre and whether such a Nox enzyme is functional. Our results show that the brain urination control centre has Nox 2 proteins, and the Nox enzyme produces a significant amount of ROS, higher than heart tissue, suggesting the importance of Nox-associated ROS production in physiology and pathology. These findings lay the groundwork for future investigation into Nox 2 and the associated oxidative damage in brain urination control centres and consequent bladder abnormalities.

**Abstract:**

Oxidative inflammatory damage to specialised brain centres may lead to dysfunction of their associated peripheral organs, such as the bladder. However, the source of reactive oxygen species (ROS) in specific brain regions that regulate bladder function is poorly understood. Of all ROS-generating enzymes, the NADPH oxidase (Nox) family produces ROS as its sole function and offers an advantage over other enzymes as a drug-targetable molecule to selectively control excessive ROS. We investigated whether the Nox 2 subtype is expressed in the micturition regulatory periaqueductal gray (PAG) and Barrington’s nucleus (pontine micturition centre, PMC) and examined Nox-derived ROS production in these structures. C57BL/6J mice were used; PAG, PMC, cardiac tissue, and aorta were isolated. Western blot determined Nox 2 expression. Lucigenin-enhanced chemiluminescence quantified real-time superoxide production. Western blot experiments demonstrated the presence of Nox 2 in PAG and PMC. There was significant NADPH-dependent superoxide production in both brain tissues, higher than that in cardiac tissue. Superoxide generation in these brain tissues was significantly suppressed by the Nox inhibitor diphenyleneiodonium (DPI) and also reduced by the Nox-2 specific inhibitor GSK2795039, comparable to aorta. These data provide the first evidence for the presence of Nox 2 and Nox-derived ROS production in micturition centres.

## 1. Introduction

Excessive production of reactive oxygen species and oxidative stress is a common pathological mechanism contributing to inflammation, tissue damage, and cell degeneration, and this leads to several highly prevalent chronic cardiovascular diseases, central nervous system (CNS) diseases, and other aging-related disorders [1,2,3]. Of all the ROS-generating enzymes, NADPH oxidase (Nox) is of particular importance. This is the only class of enzymes in the body that produce ROS as the sole product; therefore, targeting this enzyme can selectively inhibit excessive ROS production without compromising normal cellular biochemical oxidation [4,5,6]. This offers an advantage over all other enzymes that generate ROS as their by-product while performing biochemical oxidations because inhibiting these enzymes would also suppress normal biochemical transformation and hence the physiological function. The Nox family is now known to have several subtypes: Nox 1, 2, 3, 4, and 5; and Duox 1 and 2, with different tissue and cellular distributions [7]. Recent efforts in the exploration of Nox subtype-specific small-molecule inhibitors has made Nox proteins promising targetable molecules [8,9].

Oxidative stress and neuroinflammation are important pathological bases for a variety of CNS diseases [10,11]. Increasing evidence suggests that Nox enzymes contribute to oxidative damage in these conditions [12,13,14]. Various Nox subtypes have been found to be expressed in neurons, astrocytes, and microglia using in vitro cell culture and brain tissue biopsy samples [13]. Whilst Nox 4 is the first recognized Nox subtype in the CNS, and mainly participates in cell signalling but may also be involved in stroke, Nox 2 has been shown to mainly contribute to pathological damage [15,16]. The importance of Nox and associated ROS overproduction is evidenced by increased expression of Nox subtypes in experimental CNS damage and in certain pathological samples [14,15,17] and by the protective effect of interventions with small-molecule Nox inhibitors and Nox knockout [13,16,18,19,20,21,22,23,24]. However, further progress in understanding the pathological basis of Nox-derived ROS in brain damage requires knowledge of the specific distribution of Nox subtypes and associated ROS production in specialized brain regions. This is understudied, with only one publication providing evidence for subtype expression in native tissue from a small cerebral surface area [18]. However, no study has examined specialised brain structures, such as nuclei. Nox expression in specialized functional nuclei and nerve pathways will provide insight into the regional susceptibility to oxidative damage and potential targetable Nox subtypes for new therapies.

Inflammatory and oxidative damage to the CNS during aging and other chronic conditions can also lead to dysfunction in peripheral organs and tissues. Bladder overactivity or underactivity and inflammatory disorders are highly prevalent age-related chronic diseases [25,26] with many causative factors, including myogenic, neurogenic, and urothelium-based mechanisms [27]. Degenerative brain diseases are important central factors for bladder dysfunction [28,29,30,31,32,33]. The bladder storage and voiding functions are controlled by neural circuits in the brain, spinal cord, and peripheral ganglia. Periaqueductal gray (PAG) and Barrington’s nucleus (pontine micturition centre, PMC) are the main control structures for the micturition reflex [31,34,35]. When the bladder volume reaches the micturition threshold, the afferent impulses activate the micturition centre to induce a bladder contraction and relaxation of the urethra, leading to bladder voiding. During voiding, sacral parasympathetic (pelvic) nerves provide an excitatory input (cholinergic and purinergic) to the bladder and inhibitory input (nitrergic) to the urethra. Upon PMC inhibition, the urge to urinate disappears, which allows the delay of voiding until a socially acceptable place and time are found. The central PAG receives afferents from the sacral cord, which in turn sends projections to the PMC. Dysfunction in these central structures could result in bladder disorders [30]. A prerequisite for understanding the role of age-related oxidative damage in these micturition centres is whether these nuclei and regions express Nox 2, the main pathologic Nox subtype, and the ability of these two structures to produce ROS. 

We hypothesize that Nox 2 is expressed in the brain micturition centre tissues and Nox enzymes produce superoxide in these regions. In this study, we micro dissected PAG and PMC and examined the expression and function of the main pathology mediating Nox subtype (Nox 2) and the associated superoxide production.

## 2. Materials and Methods

### 2.1. Animal Tissue Preparations

Male C57BL/6J mice (2–5 months) were used as the experimental model and were obtained from Charles River and maintained under barrier-fed and specific pathogen-free conditions with a standard balanced diet and free access to water, at a temperature of 20–24 °C and humidity of 45 to 65%. Mice were humanely culled by a schedule-1 procedure in accordance with the United Kingdom Animals (Scientific Procedures) Act of 1986. All experiments were approved by the local ethics committee and UK home office.

The brain, heart, and aorta were promptly removed after culling and collected in cold physiological saline (artificial cerebral spinal fluid, ACSF; see the next section). According to *Paxinos and Franklin’s Mouse in Stereotaxic Compacts—4th edition*, the brain regions of the PAG and PMC in mice were dissected using a Rodent Brain Slicer Matrix (Zivic instruments, Pittsburgh, PA, USA) with 0.5 mm coronal slice intervals, under microscopic guidance. All areas were approximately 500 µm × 1000 µm to ensure that the specialized area was included (see Table 1). The muscular layer was removed from the aorta and the intima layer was used for the experiment. A small piece (approximately 5 mg) of myocardium was taken from the left ventricular apex.

### 2.2. Lucigenin-Enhanced Chemiluminescence

Real-time NADPH-dependent superoxide production in the brain, heart tissue, and aorta was measured using lucigenin-enhanced chemiluminescence by a luminescence plate reader (BMG Lumistar, Ortenberg Germany). In this method, superoxide anion, the body’s primary form of reactive oxygen species, is measured by chemiluminescence generated in the presence of lucigenin [36]. The tissue samples (2–4 mg) were placed in the sample wells in an opaque 96-well plate (Costor, Corning Incorporated, Kennebunk, ME, USA) and each well was filled with a final volume of 200 µL of artificial cerebral spinal fluid (ACSF). ACSF contained 128 mM NaCl, 3 mM KCl, 1 mM MgCl_2_·6H_2_O, 24 mM NaHCO_3_, 0.5 mM NaH_2_PO_4_·2H_2_O, 30 mM glucose, and 1.5 mM CaCl_2_. The solution was bubbled with 95%O_2_ + 5%CO_2_ for 1 h at 37 °C prior to experimentation. The basal luminescence signal was recorded in the presence of 5 µM lucigenin, and the NADPH-stimulated luminescence signal was measured with the addition of 100 µM NADPH, a cofactor of Nox enzymes. Tiron (50 mM), a superoxide scavenger, was applied to validate the superoxide production. Measurements were taken at 37 °C, the optimal sampling time per well determined by the instrument, and a total of 56 cycles were recorded. The quantity of photons collected was expressed as relative light units (RLUs) per unit tissue weight (RLU/mg). 

### 2.3. Western Blot

Isolated brain and heart tissues were homogenized in liquid nitrogen using a pre-cooled pestle and mortar. The homogenate powder was lysed in an appropriate volume of RIPA buffer and sonicated at 100 Amp using a VC130PB ultrasonic processor (Sonics & Materials Inc., Newtown, CT, USA) for 30 s to release intracellular components. Samples were centrifuged (13,000 rcf, 30 s, 4 °C) and the protein concentration of the supernatants determined using a colorimetric protein assay kit (DC protein assay, BioRad, Watford, UK). Supernatants were then mixed in a 3:1 ratio with protein loading buffer (LI-COR, Bad Homburg, Germany) and incubated at 95 °C for 5 min to fully dissolve the lysate. HEK293 cells were used as a positive control for antibody binding. Tissue lysates (15 μg) were loaded and the proteins electrophoretically separated on precast gels (8–16% Mini-PROTEAN TGX, BioRad, Watford, UK) and then transferred to a 0.45 μm nitrocellulose membrane (Novex, Thermo Fisher, Waltham, MA, USA) using a BioRad blotting system. Membranes were probed with the primary antibodies at 4 °C overnight. The primary antibodies used were NOX 2/gp91 phox (Rabbit, ab129068, Abcam, Cambridge, UK, 1:500) and β-actin (Mouse, 8H10D10, New England Biolabs, Ipswich, MA, USA, 1:4500). The protein under study bound by the primary antibody was detected using secondary antibodies conjugated with Infra-red dyes: Goat anti-Rabbit IgG IRDye^®^800CW (926-32211, LI-COR, Bad Homburg, Germany, 1:3000) and Donkey anti-Mouse IgG IRDye^®^680RD (926-68072, LI-COR, Bad Homburg, Germany, 1:3000). The signal was detected and analysed using an Odyssey CLx infrared imaging system (LI-COR, Bad Homburg Germany). Each membrane was probed for β-actin (New England Biolabs, Ipswich, MA, USA) to serve as a loading control. 

### 2.4. Data Analysis

Data are expressed as mean ± SEM. For data with a normal distribution, the difference between two group means was tested with Student’s *t*-test, paired or unpaired as appropriate. The null hypothesis was rejected at *p* < 0.05. For data that did not follow a normal distribution or had an unknown distribution, the difference between two group means was tested with equivalent non-parametric tests. The difference among multiple means was tested with ANOVA followed by pair-wise comparisons or non-parametric equivalents. Statistical analysis was performed using GraphPad Prism (version 8.4.3, GraphPad Software, San Diego, CA, USA).

## 3. Results

### 3.1. Identification of Nox 2 in PAG and PMC

**Figure 1:**
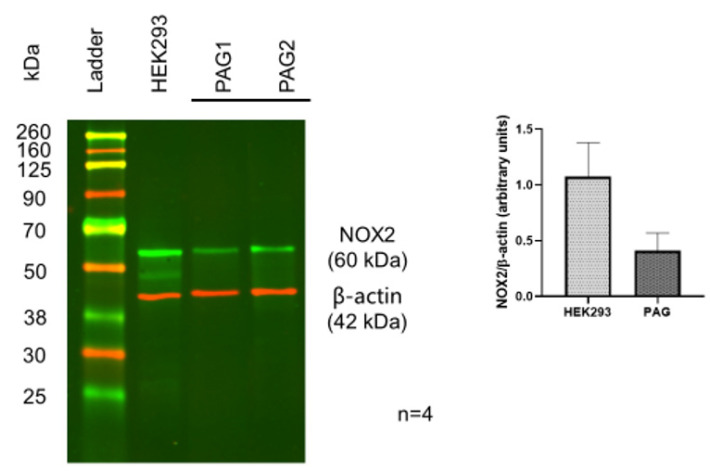
Western blot of Nox-2 in PAG from the mouse brain. HEK293 cells served as a positive control. Data show positive expression of Nox-2 in PAG, comparable to that in HEK293 cells.

**Figure 2:**
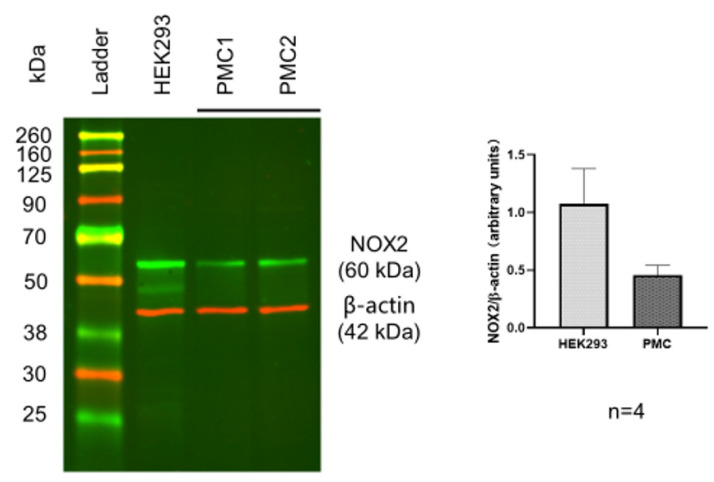
Western blot of Nox-2 in PMC from the mouse brain. HEK293 served as a positive control. Data show positive expression of Nox-2 in PMC, comparable to that in HEK293 cells.

To identify the existence of Nox 2 molecules in PAG and PMC, Western blots with a Nox 2-specific antibody were performed. The Western blot results showed a clear protein band with the expected molecular weight of Nox 2 from the PAG extracts. This was validated by Nox 2-positive HEK 293 cells with the same molecular weight and comparable intensity, confirming significant expression of Nox 2 in the PAG (Figure 1, *n* = 4). Similar Nox 2-positive staining was also observed in the PMC tissue (Figure 2, *n* = 4). These data show that Nox 2 proteins are expressed in PAG and PMC, with relatively high expression, and the expression in PAG and PMC is similar. These Nox proteins serve as the molecular basis of Nox-derived ROS production. 

### 3.2. NADPH-Dependent Superoxide Production in PAG and PMC

**Figure 3:**
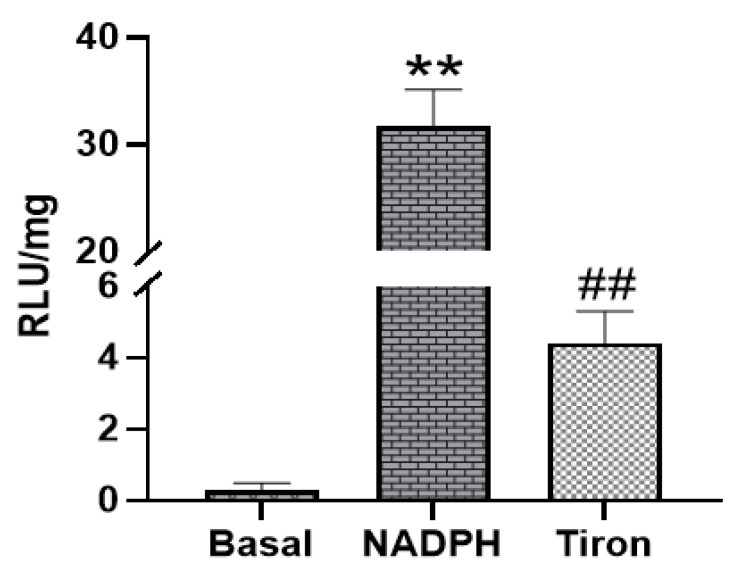
Superoxide production measured by lucigenin-enhanced luminescence in PAG tissue. Addition of NADPH stimulated high levels of superoxide production; ** *p* < 0.01 vs. basal; ^##^ *p* < 0.01 vs. NADPH, *n* = 21.

**Figure 4:**
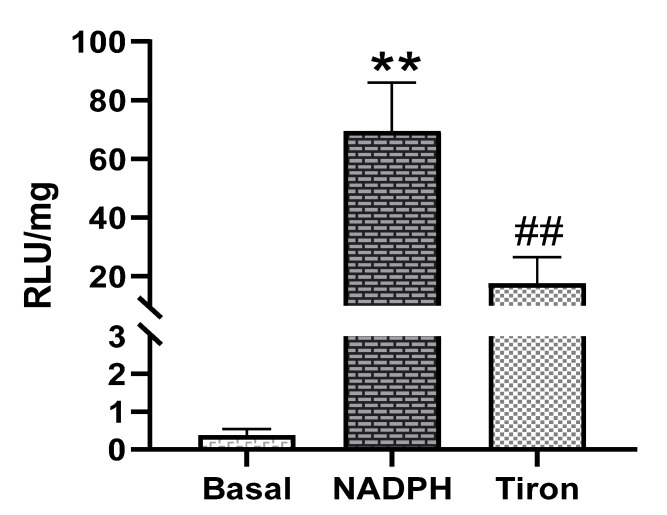
Superoxide production measured by lucigenin-enhanced luminescence in PMC tissue. Note: high levels of superoxide production stimulated by NADPH ** *p* < 0.01 vs. basal; ^##^ *p* < 0.01 vs. NADPH, *n* = 21.

**Figure 5:**
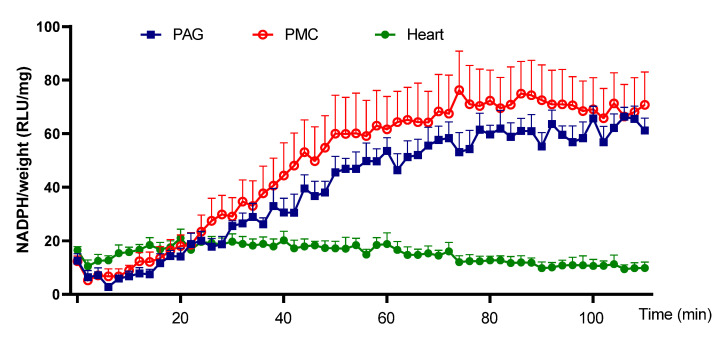
Time course of superoxide generation in PAG, PMC, and cardiac tissue. The plot shows a much more sustained increase of superoxide in brain tissues PAG and PMC than that in cardiac tissue. *n* = 6.

Next, the ability of PAG and PMC tissues to produce Nox-derived superoxide was determined. NADPH addition to the sample wells stimulated a large increase of the luminescence intensity from the basal level signals in PAG and PMC tissues (Figure 3 and Figure 4). The real-time response to NADPH in PAG and PMC exhibited a sustained increase of the signal for a longer duration than in heart tissue (Figure 5). The specificity of superoxide was confirmed by its sensitivity to the superoxide scavenger Tiron (50 mM; Figure 3 and Figure 4). 

#### Comparison of Superoxide Production from PAG and PMC with That in Myocardium

**Figure 6:**
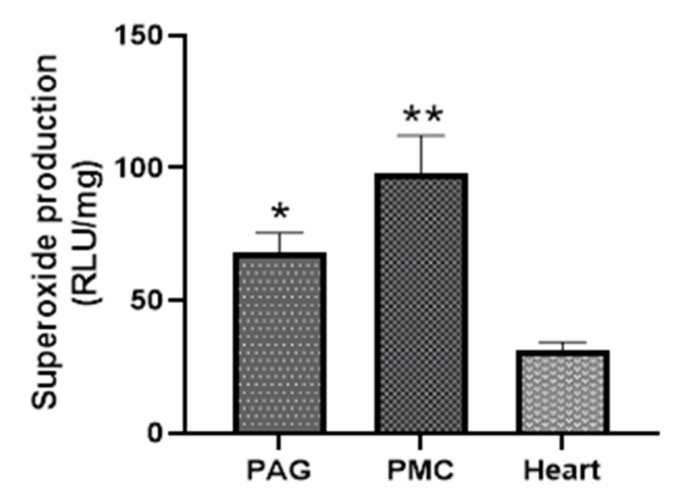
Comparison of superoxide production from PAG and PMC with that in myocardium. * *p* < 0.05, ** *p* < 0.01 vs. heart, *n* = 11.

The strength of Nox-derived superoxide production in PAG and PMC tissues was determined by comparison with that in cardiac muscle tissue. Figure 6 shows that PAG and PMC produced higher levels of NADPH-dependent superoxide than that in the myocardium. The kinetic data also exhibited a much more sustained response in PAG and PMC compared with myocardium (Figure 5).

### 3.3. Inhibition of NADPH-Dependent Superoxide Production by Nox Inhibitors

**Figure 7:**
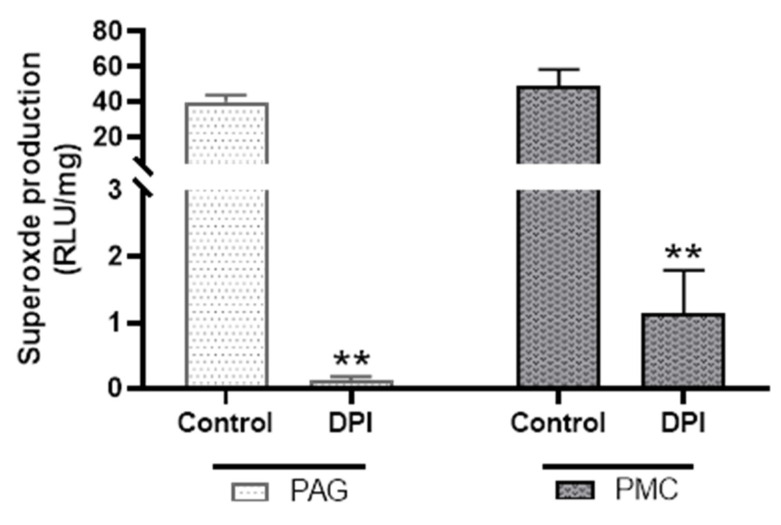
Effect of diphenyleneiodonium (DPI) on superoxide production in PAG and PMC tissues. DPI significantly reduced superoxide production in both brain regions. *n* = 6; ** *p* < 0.01 for both PAG and PMC groups.

To determine how much of the NADPH-dependent superoxide production in PAG and PMC tissues was from Nox enzymes, diphenyleneiodonium (DPI), a broad-spectrum inhibitor of Nox activities, was used. DPI (20 µM) reduced NADPH-dependent superoxide production by more than 90% in both PAG and PMC (Figure 7). These data suggest that the main source of superoxide generation in PAG and PMC is Nox enzymes. 

**Figure 8:**
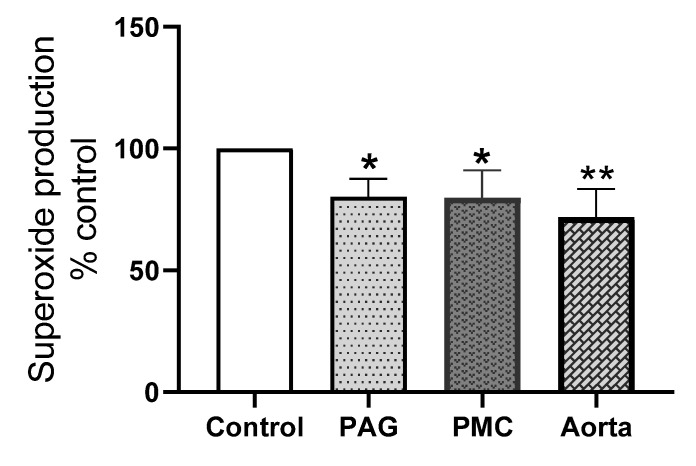
Effect of GSK2795039 on superoxide production in PAG, PMC, and aorta. GSK2795039 significantly reduced NADPH-dependent superoxide production in PAG and PMC, comparable to that in the aorta. Note: the level of superoxide without the inhibitor in each tissue was taken as 100%. * *p* < 0.05 vs. control, PAG, *n* = 10; PMC, *n* = 6; ** *p* < 0.01 vs. control, aorta, *n* = 6. GSK2795039 concentration: 25 μM.

To further dissect the contribution of the Nox 2 subtype to superoxide production in these brain tissues, the effect of the Nox 2-specific inhibitor, GSK2795039 was examined. The efficacy of GSK2795039 has only been assessed in cell-free enzyme preparations and on PMA and iron-stimulated ROS release in intact cells while its effect on endogenous superoxide release from cells or tissue has not been tested [37,38]. We therefore used aortic tissue, known to produce superoxide mainly from Nox 2, for the comparison. Figure 8 shows that GSK2795039 caused a significant reduction of superoxide generation in both PAG and PMC and in the aorta and there was no statistical difference between the inhibition in these brain tissues and the aorta. The average reduction induced by GSK2795039 in the aorta was 28% while the inhibition in PAG and PMC was about 20%. This shows that GSK2795039-induced inhibition of superoxide production in PAG and PMC is over 2/3 of the inhibition in aorta. This suggests that Nox 2 is a main contributor to Nox-derived superoxide in these brain regions.

## 4. Discussion

In this study, we micro dissected the specialised brain regions PAG and PMC to investigate Nox expression and the source of superoxide. These results show that Nox 2 is expressed in PAG and PMC, and these brain regions produce a significant amount of NADPH-dependent superoxide. Furthermore, the source of superoxide is mainly from Nox enzymes and partly from Nox 2. To our knowledge, this is the first study that shows Nox 2 subtype expression and Nox-derived superoxide production in these micturition regulatory structures in the CNS. It is also the first report on the Nox subtype location and its activity in any specialised brain nucleus. This suggests that the Nox system could exist in another brain nucleus.

Nox 2 has been shown to mainly mediate pathological processes in the brain and can be considered as a pathological Nox subtype [13]. The fact that PAG and PMC express this Nox subtype in high abundance suggests that these micturition centres could be subjected to oxidative damage due to Nox 2 activity. Nox 2 has thus been identified to be the molecular basis for ROS production and potential oxidative damage in these brain regions. This may have important pathological implications, as oxidative damage to these structures as a result of increased Nox 2 activity could disrupt normal central control of the micturition reflex and cause voiding disorders. Indeed, in several other chronic brain disorders, Nox 2 has been suggested to contribute to inflammation and oxidative damage [14,15,17]. This warrants further investigation into the role of Nox 2 in these brain regions in neurogenic bladder dysfunctions. Our finding also identifies Nox 2 as a potential drug-targetable molecule for precision therapy in the future. There is currently intense interest in developing subtype-specific Nox inhibitors, and Nox 2 inhibitors are well sought-after [7,8]. This may lead to more specific drug therapies for controlling ROS-mediated brain damage and associated peripheral organ dysfunction, such as bladder diseases. 

The magnitude of the NADPH-dependent superoxide production emphasises the significance of the ROS-generating ability of these two brain regions. This is consolidated by data showing significantly higher levels of superoxide production in these brain tissues than that in cardiac tissue. Cardiac muscle is known to generate high levels of ROS and be susceptible to ROS damage [4,39,40]. The profound inhibition by the Nox inhibitor DPI shows that the main source of superoxide is from Nox enzymes in these brain regions. Furthermore, we evaluated the specific contribution from Nox 2. As the efficacy of GSK2795039 on endogenous superoxide generation has not been assessed in intact tissue without exogeneous stimuli, we included aorta as a positive control for Nox 2 production [41]. Aorta is known to mainly express Nox 2, and Nox 2 activity contributes to the main part of superoxide production [41]. Data show that a significant portion of the superoxide production in PAG and PMC is from Nox 2 as evidenced by the similar levels of inhibition in these brain regions and aorta tissue by this Nox 2 specific inhibitor. These biochemical characteristics of Nox activity in the brain regions underpin a key role for Nox-derived ROS in the contribution to oxidative stress in the CNS. Its role in brain damage-associated mechanisms for bladder dysfunction deserves further investigation. There is evidence for increased ROS production in these brain regions in some brain disorders [14,42,43,44]. The source of superoxide could be Nox enzymes as supported by the Nox predominance as the superoxide source from our findings. NADPH-dependent superoxide production has been reported in bladder urothelium and may lead to oxidative damage and contribute to bladder overactivity [27]. Furthermore, superoxide generation associated with Nox activity can be enhanced by TRPV4 receptor activators, suggesting additional modulation of ROS generation by sensory receptors and inflammatory ligands. This implies that ROS overproduction and oxidative stress could serve as a pathological mechanism of tissue damage at both central and peripheral levels. Several CNS degenerative diseases have been shown to contribute to bladder dysfunction, where oxidative damage and inflammation serve as a mechanism of cell and tissue damage [28,29,30,31,32,33]. How Nox-derived ROS changes in these conditions and the consequent bladder damage awaits further investigation.

Our real-time monitoring of superoxide production in live tissue also revealed interesting dynamics of Nox-derived superoxide production in the PAG and PMC. ROS production in these tissues is persistent and much more sustained than that from cardiac muscle tissue. This implies that Nox-derived ROS in these brain regions, once initiated, produces long-lasting oxidative damage to the tissue. This brain region-specific mode of action of Nox-driven superoxide production could serve as a molecular mechanism for chronic damage to these brain regions.

Increased ROS production and oxidative stress is a common pathology-related process during aging. This is supported by data from both humans and animal models during aging and in many aging-associated diseases [2,45,46,47]. The characteristics of the Nox and ROS production identified in PAG and PMC suggest that the Nox system could drive aging-related ROS upregulation and oxidative damage. The pathological role of Nox-derived ROS in age-related neurogenic bladder dysfunction due to oxidative damage merits further study. 

In summary, this study provides the first evidence for Nox 2 expression and Nox-derived ROS in CNS micturition centres. The predominance of this system and its characteristics revealed by this study lay the groundwork for future study into the mechanistic basis of aging and inflammation-associated neurogenic bladder disorders.

## 5. Conclusions

Results from this study provide the first evidence for the presence of the pathologically important Nox 2 subtype in PAG and PMC related to central control of micturition and the high capability of both brain micturition regions to produce Nox-derived superoxide, supported by the inhibition of the broad-spectrum Nox 2 inhibitor DPI and the sustained nature of such ROS production by comparison with cardiac tissue known to produce high levels of ROS. A significant portion of Nox-derived superoxide production is from Nox 2 enzyme, verified by the effect of the Nox 2-specific inhibitor GSK2795039, comparable to the Nox 2-predominant tissue, the aorta. The predominance of this system and its characteristics revealed by this study lay the groundwork for future study investigating the mechanistic basis of aging and inflammation-associated neurogenic bladder disorders. 

## Figures and Tables

**Table 1:** Dissection of specialized brain regions.

Area	To Bregma (mm)		Location (Brain Figure)	
	From	To	From	To
PAG	−2.79	−3.15	54	57
PMC	−5.33	−5.63	75	78

## Data Availability

Data is contained within the article.
